# A retrospective evaluation of Bayesian-penalized likelihood reconstruction for [^15^O]H_2_O myocardial perfusion imaging

**DOI:** 10.1007/s12350-022-03164-5

**Published:** 2023-01-19

**Authors:** Reetta Siekkinen, Chunlei Han, Teemu Maaniitty, Mika Teräs, Juhani Knuuti, Antti Saraste, Jarmo Teuho

**Affiliations:** 1 Turku PET Centre, Turku University Hospital, Kiinamyllynkatu 4-8, 20520 Turku, Finland; 2 Turku PET Centre, University of Turku, Turku, Finland; 3 Department of Medical Physics, Turku University Hospital, Turku, Finland; 4 Heart Centre, Turku University Hospital, Turku, Finland; 5 Institute of Biomedicine, University of Turku, Turku, Finland

**Keywords:** CAD, myocardial ischemia and infarction, PET, MPI, myocardial blood flow, hybrid imaging

## Abstract

**Background:**

New Block-Sequential-Regularized-Expectation-Maximization (BSREM) image reconstruction technique has been introduced for clinical use mainly for oncologic use. Accurate and quantitative image reconstruction is essential in myocardial perfusion imaging with positron emission tomography (PET) as it utilizes absolute quantitation of myocardial blood flow (MBF). The aim of the study was to evaluate BSREM reconstruction for quantitation in patients with suspected coronary artery disease (CAD).

**Methods and Results:**

We analyzed cardiac [^15^O]H_2_O PET studies of 177 patients evaluated for CAD. Differences between BSREM and Ordered-Subset-Expectation-Maximization with Time-Of-Flight (TOF) and Point-Spread-Function (PSF) modeling (OSEM-TOF-PSF) in terms of MBF, perfusable tissue fraction, and vascular volume fraction were measured. Classification of ischemia was assessed between the algorithms. OSEM-TOF-PSF and BSREM provided similar global stress MBF in patients with ischemia (1.84 ± 0.21 g⋅ml^−1^⋅min^−1^ vs 1.86 ± 0.21 g⋅ml^−1^⋅min^−1^) and no ischemia (3.26 ± 0.34 g⋅ml^−1^⋅min^−1^ vs 3.28 ± 0.34 g⋅ml^−1^⋅min^−1^). Global resting MBF was also similar (0.97 ± 0.12 g⋅ml^−1^⋅min^−1^ and 1.12 ± 0.06 g⋅ml^−1^⋅min^−1^). The largest mean relative difference in MBF values was 7%. Presence of myocardial ischemia was classified concordantly in 99% of patients using OSEM-TOF-PSF and BSREM reconstructions

**Conclusion:**

OSEM-TOF-PSF and BSREM image reconstructions produce similar MBF values and diagnosis of myocardial ischemia in patients undergoing [^15^O]H_2_O PET due to suspected obstructive coronary artery disease

**Supplementary Information:**

The online version contains supplementary material available at 10.1007/s12350-022-03164-5.

## Introduction

Myocardial perfusion imaging (MPI) with Positron emission tomography (PET) can be used as a first line diagnostic test in patients with suspected obstructive coronary artery disease (CAD).^1^ MPI PET allows to quantitate myocardial blood flow (MBF), perfusable tissue fraction (PTF), and vascular volume fraction (VL).^2–4^ Furthermore, the quantitative MBF during vasodilator stress is a key factor determining myocardial ischemia when compared with intracoronary fractional flow reserve.^5–7^ However, such a clinical interpretation can only be made if the PET image reconstruction techniques provide accurate image quantification.^8^ Therefore, whenever a new clinical reconstruction technique is introduced, it should be compared against the de facto state-of-the-art reconstruction technique.^9^

Recently, a new reconstruction algorithm Block-Sequential-Regularized-Expectation-Maximization (BSREM, vendor name: Q.Clear) was implemented in clinical use.^10^ So far, investigations with BSREM have been mainly conducted with [^18^F]FDG^11–15^ in oncology studies, in which sensitive detection of tumors and their metastases is the main focus. However, there are only a few studies where BSREM is applied in dynamic perfusion studies, in which absolute quantitation and detection of ischemia is the main task. Evaluations have been performed with [^13^N]NH_3_ as part of sarcoidosis diagnostics, with analysis of rest perfusion in 21 patients.^16^ Very recently, Nordström et al. thoroughly investigated the effect of various reconstruction parameters with [^15^O]H_2_O, including BSREM.^17^ The authors reported no significant change in diagnosis but prompted that the findings to be confirmed with a larger number of patients. As MPI PET is nowadays more increasingly used for detection of myocardial ischemia, the impact of BSREM should be investigated in patients with suspected CAD, both at rest and during stress and using various perfusion tracers and its effect on diagnosis should be confirmed.

Previous studies have indicated that physical test objects (phantoms) are practical when evaluating the accuracy of quantitative values derived from PET images against a ground-truth measurement.^18,19^ Our group has recently assessed the accuracy of flow measurements in [^15^O]H_2_O MPI PET, using a dynamic PET phantom.^20^ In the study, comparison of an Ordered-Subset-Expectation-Maximization with Time-of-Flight and Point-Spread Function modeling (OSEM-TOF-PSF) algorithm and other reconstruction methods, i.e., the BSREM algorithm, resulted in image-derived flow differing 7% at maximum in comparison to the reference flow.^20^

The goal of the present study was to evaluate the accuracy of BSREM quantification of myocardial perfusion and the impact on the classification of ischemia in a large group of patients with suspected CAD. The BSREM was compared to well-established state-of-the-art reconstruction OSEM-TOF-PSF technique.

## Materials and methods

Data of 179 subjects undergone stress or rest-stress [^15^O]H_2_O myocardial perfusion imaging due to suspected obstructive coronary artery disease in Turku PET Centre (Turku, Finland) were analyzed retrospectively. The cohort consisted of 63 females and 116 males ageing (mean ± SD) 66 ± 10 years. Their body mass index was 30 ± 6 kg⋅m^−2^. There were 75 current or previous smokers, 35 were diabetics and 98 had hypertension. In 17 patients, rest and stress imaging was performed, and 162 had undergone only stress imaging. The Hospital District of Southwest Finland granted permission for the retrospective study (number T93/2019). Due to the retrospective nature of the study, the collection of informed consents from individual subjects was waived.

All subject data was acquired with a digital Discovery MI PET/CT system (DMI-20, GE Healthcare, Milwaukee, US). The DMI-20 PET detector system consists of four detector rings and one ring comprises 136 detector blocks. Each block employs 3 × 6 array of silicon photomultiplier (SiPM) detectors with a 4 × 9 array of lutetium-yttrium oxyorthosilicate (LYSO) crystals with one crystal element size of 3.95 mm × 5.3 mm × 25 mm. The axial and transaxial FOV sizes of the DMI-20 are 20 cm and 70 cm, respectively. The coincidence timing and energy windows are 4.9 ns and 425-650 keV. The system performance details are described in Hsu et al.^21^

Imaging protocol included a CT acquisition for attenuation correction (CTAC) followed by a dynamic PET study with [^15^O]H_2_O. The CTAC acquisition parameters were as follows: tube voltage of 120 kV, tube current of 120 mA and noise index of 30.00. The helical full rotation time was 0.5 seconds, while helical thickness was set at 3.75 mm with pitch of 1.375:1 and speed 55 mm/rotation. The CTAC was reconstructed using full 70 cm field-of-view (FOV).

All 179 subjects had undergone dynamic adenosine stress [^15^O]H_2_O PET. Also, 17 subjects had rest PET prior to stress PET. The PET protocol for both rest and stress imaging was as previously reported.^22,23^ After allowing radioactivity from the rest PET imaging injection to decay, adenosine infusion was started 2 minutes before the start of the stress PET acquisition, and was infused 140 µg per kilogram of body weight per minute. Patients were injected with 500 MBq bolus of [^15^O]H_2_O from an automatic dispenser (Hidex Oy, Finland), and PET acquisition was started 25 seconds after the bolus injection. PET acquisition lasted 4 minutes and 40 seconds and the data was thereafter binned into dynamic frame lengths of 14 × 5 seconds, 3 × 10 seconds, 3 × 20 seconds and 4 × 30 seconds.

Two reconstructions for PET data were applied. The first was the three-dimensional OSEM algorithm with TOF and PSF (OSEM-TOF-PSF) (vendor name: VPFX-S) with 3 iterations and 16 subsets. The reconstructed PET FOV was set to 35 cm, with an image matrix of 192 × 192 and 5.0 mm post filter. The BSREM reconstructions (vendor name: Q.Clear) were performed with a FOV of 35 cm and matrix size of 192 × 192. In the Discovery MI PET/CT, the beta value of BSREM is set to a value of 350 by default, which we did not modify for this study. The beta value 350 was selected as recommended by the manufacturer.^24^ All PET reconstructions were performed using the clinical software installed on the PET/CT system, with software version of pet_col_bb.31.

Of the 179 subjects undergone only stress imaging, two subjects had to be excluded, one due to a failed injection and the other due to missing frames on the BSREM reconstruction. The final dataset consisted of 177 subjects.

All image analysis with quantification was performed using Carimas 2.10 (Turku PET Centre, Finland) by a single observer (CH) with over 20 years of experience in MPI PET image analysis. For comparison of two reconstruction algorithms OSEM-TOF-PSF and BSREM, myocardial segmentation was performed by using similar volumes of interest (VOI). The delineation and corresponding segmentation of the left ventricle was first performed for the OSEM-TOF-PSF reconstructed images after which the VOIs were copied to BSREM reconstructed images without modification. From each VOI, the time-activity curves (TACs) for the input function and myocardial tissue were extracted.

Kinetic modeling designed for [^15^O]H_2_O tracer was performed based on the image-derived TACs and by using the model presented by Iida H. et al.^2–4^ which is implemented in Carimas 2.10. In addition to MBF (in units of ml⋅g^−1^⋅min^−1^), the perfusable tissue fraction (PTF, in units of ml⋅ml^−1^) and vascular volume fraction (VL in units of ml⋅ml^−1^) were modelled in order to analyze the effect on other quantitative parameters of kinetic modeling, too. The default parameters used for kinetic modeling were as follows: initial guess for MBF, PTF, and VL was 0.5, for partition coefficient 0.9464 and for beta value 0.93. Fitting of the TACs was performed using uniform weighting. Finally, the MBF values were visually analyzed in standard 17- and 3-segment polar maps, and all parameters were exported to Excel spreadsheets.^25^ The 17-segment polar maps represent the standard myocardial segments defined by American Heart Association,^25^ and the 3-segment polar map represent the standard myocardial territories of the left anterior descending coronary artery (LAD), the left circumflex coronary artery (LCX) and the right coronary artery (RCA).

The spreadsheets containing MBF, PTF, and VL values from the 17- and 3-segments were imported to MATLAB version 2020a (Mathworks Inc. Natick, US). Classification and analysis of subjects was performed automatically using in-house developed software pipeline in MATLAB 2020a. The analysis pipeline consisted of automatic classification of patients to ischemic and non-ischemic groups, followed by visual and statistical analysis. Myocardial ischemia was defined in the presence of stress MBF < 2.3 ml⋅g^−1^⋅min^−1^ in at least two neighboring segments as previously validated.^5^ Segments 2, 3, and 17 were excluded from the analysis due to the presence of the fibrotic area of the basal septum in segments 2 and 3, and potential uncertainties in VOI definition in segment 17. In the 3-vessel analysis, ischemia was defined if average stress MBF was < 2.3 ml⋅g^−1^⋅min^−1^ in one of the vessel territories.

For both reconstructions, we measured mean and standard deviation (SD) values from MBF, PTF, and VL over all segments despite whether the segment was ischemic or non-ischemic. Thereafter, we calculated the mean values of the parameters over the groups of patients classified as ischemic and non-ischemic as well as over the rest patients. The results are presented as mean and SD values for each group, as well as the minimum and maximum value per group. Similarly, we measured the mean relative differences between OSEM-TOF-PSF and BSREM as well as SD of mean relative differences per each patient group. MBF values and the relative differences of MBF values are visualized in boxplots for the whole cohort. In addition, we represent regression plots and Bland–Altman plots in order to determine the correlation of MBF, PTF, and VL between OSEM-TOF-PSF and BSREM. Also, statistical testing between MBF, PTF, and VL values calculated from OSEM-TOF-PSF and BSREM reconstructed images was performed. A two-tailed *t*-test was applied to indicate if there are significant differences in MBF, PTF and VL with different reconstruction algorithms, using *P* < 0.05 as the significance threshold. Statistical comparison was performed for each segment over all patients. Bonferroni–Holm correction with α = 0.05 was applied to account for multiple comparisons.

## Results

Table 1 presents the mean, SD and range (from minimum to maximum) values measured from subjects classified as ischemic, non-ischemic as well as from the rest subjects for the 17- and 3-segment polar maps. Also, the mean relative differences and SD of differences measured between OSEM-TOF-PSF and BSREM are presented for each patient group. The measured MBF, PTF and VL values are similar (*P* > .5) between OSEM-TOF-PSF and BSREM. The absolute differences are smaller than 0.5 ml⋅g^−1^⋅min^−1^, 0.1, and 0.1, respectively. The relative mean differences between OSEM-TOF-PSF and BSREM are smaller than 4%, 2%, and 6% for stress studies (non-ischemic and ischemic) and 7%, 4% and 6% for rest studies, for MBF, PTF, and VL in Table 1.

**Table 1:** MBF, PTF, and VL derived by kinetic modeling presented as mean, standard deviation (SD), minimum, and maximum values as well as relative differences between OSEM-TOF-PSF and BSREM at 17 segments and 3 segments

	17 segments		3 segments	
	OSEM Mean ± SD (Min − max)	BSREM Mean ± SD (Min − max)	OSEM Mean ± SD (Min − max)	BSREM Mean ± SD (Min − max)
	Mean diff (%) ± SD diff (%)		Mean diff (%) ± SD diff (%)	
	MBF (ml⋅g^−1^⋅min^−1^)			
Ischemic	1.84 ± 0.21	1.86 ± 0.21	1.85 ± 0.06	1.86 ± 0.07
	(1.34–2.11)	(1.35–2.14)	(1.71–2)	(1.72–2.04)
	3.18% ± 2.97%		2.24% ± 2.03%	
Non-ischemic	3.26 ± 0.34	3.28 ± 0.34	3.31 ± 0.17	3.33 ± 0.17
	(2.47–3.75)	(2.51–3.78)	(3.15–3.53)	(3.17–3.56)
	3.32% ± 2.13%		2.54% ± 2%	
Rest	0.97 ± 0.12	1 ± 0.13	1.12 ± 0.06	1.15 ± 0.1
	(0.77–1.15)	(0.8–1.18)	(1.05–1.18)	(1.04–1.26)
	6.59% ± 3.58%		3.07% ± 2.61%	

	PTF			
Ischemic	0.77 ± 0.06	0.77 ± 0.06	0.76 ± 0.04	0.77 ± 0.04
	(0.67–0.86)	(0.66–0.87)	(0.72–0.8)	(0.72–0.81)
	1.84% ± 1.73%		1.31% ± 1.15%	
Non-ischemic	0.76 ± 0.06	0.77 ± 0.07	0.76 ± 0.04	0.77 ± 0.04
	(0.65–0.87)	(0.65–0.88)	(0.71–0.81)	(0.71–0.81)
	2.13% ± 1.83%		1.76% ± 1.38%	
Rest	0.72 ± 0.04	0.71 ± 0.04	0.72 ± 0.02	0.72 ± 0.02
	(0.65–0.78)	(0.65–0.77)	(0.7–0.74)	(0.7–0.74)
	3.58% ± 6.44%		2.07% ± 2.72%	

	VL			
Ischemic	0.22 ± 0.06	0.21 ± 0.06	0.21 ± 0.04	0.21 ± 0.04
	(0.13–0.36)	(0.13–0.35)	(0.17–0.27)	(0.16–0.26)
	5.49% ± 4.99%		3.8% ± 3.22%	
Non-ischemic	0.24 ± 0.06	0.24 ± 0.06	0.23 ± 0.04	0.23 ± 0.04
	(0.14–0.37)	(0.14–0.36)	(0.23–0.29)	(0.19–0.28)
	5.86% ± 5.86%		3.79% ± 3.09%	
Rest	0.22 ± 0.06	0.22 ± 0.05	0.22 ± 0.03	0.22 ± 0.03
	(0.15–0.34)	(0.14–0.34)	(0.19–0.25)	(0.18–0.25)
	5.15% ± 5.17%		3.66% ± 2.49%	

There were no significant differences in any parameters between the reconstruction methods (*P* > .05)

**Figure 1:**
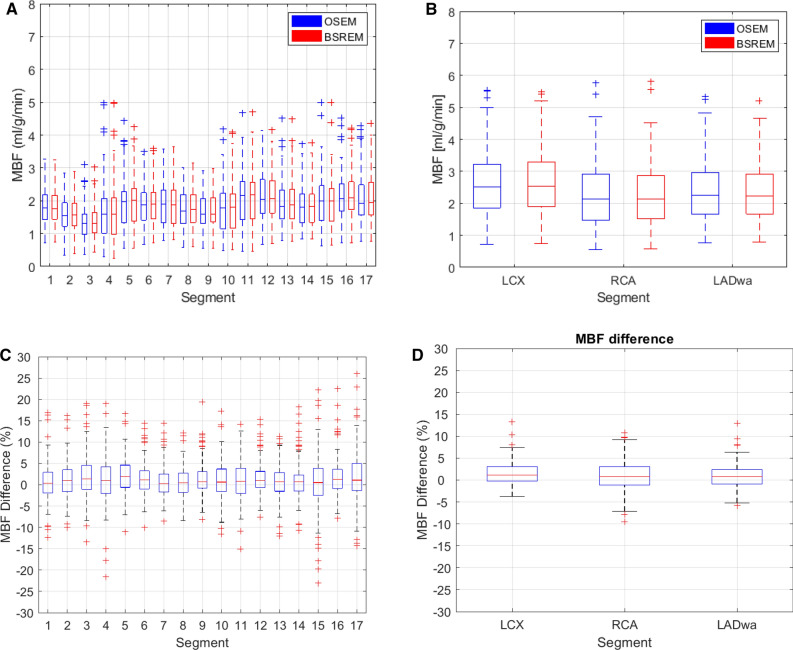
Boxplots of relative differences (**a**, **b**) and absolute values (**c**, **d**) of segmental MBF in whole patient group between OSEM-TOF-PSF and BSREM presented for the **a**, **c**) 17- and **b**, **d**) 3-segment polar maps

Figure 1 presents the boxplots of relative differences between OSEM-TOF-PSF and BSREM (a, b) and the absolute values (c, d) of segmental MBFs from the 17- (Figure 1a, c) and 3-segment (Figure 1b, c) polar maps for the whole patient cohort. The segmental boxplots show that the median MBF differences are near zero and the quantiles of MBF differences are smaller than 5%. Similarly, the absolute values show no clear difference between the reconstructions for any segment (Figure 1c, d). Also, there is no clear difference between the 3- and 17-segmental boxplots (Figure 1a, b).

**Figure 2:**
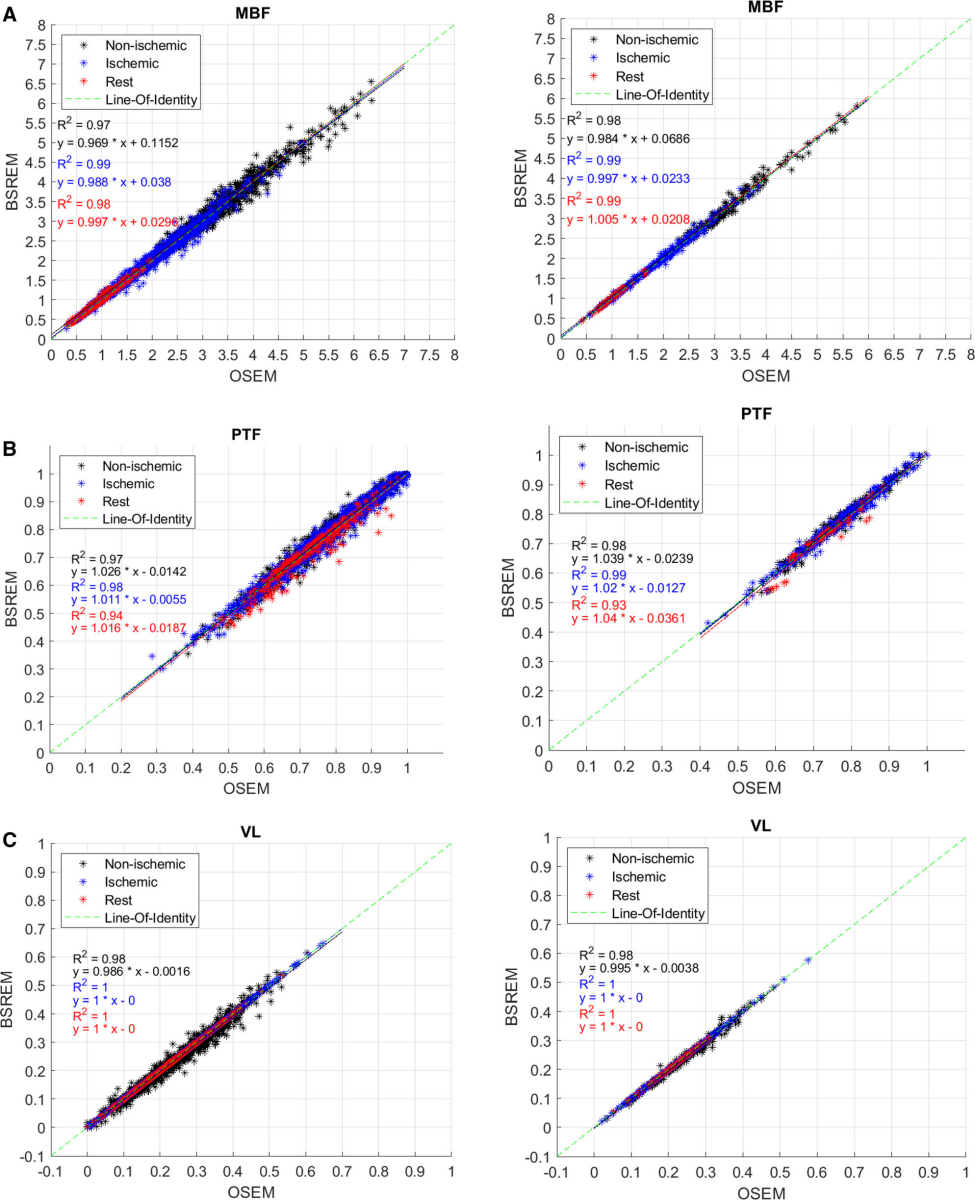
Correlation analysis of segmental **a** MBF (ml⋅g^−1^⋅min^−1^), **b** PTF and **c** VL obtained using OSEM-TOF-PSF and BSREM reconstructions. Segments of non-ischemic (black) and ischemic (blue) patients as well as rest (red) studies are shown in different colors. The line-of-identity is visualized in green. Results for 17 segments are shown in the left column and 3 segments (LAD, RCA, and LCX) in the right column

Figure 2 shows the correlation analysis for segmental MBF, PTF, and VL obtained using OSEM-TOF-PSF and BSREM reconstructions. The *R*^2^ values of the regression lines were larger than 0.97, 0.97 and 0.98, for stress studies (non-ischemic and ischemic) and 0.98, 0.93, and 1.0 for rest studies, for MBF, PTF, and VL for both 17- and 3-segments. The intercept of the regression line is very close to zero for all parameters and segments (Figure 2).

**Figure 3:**
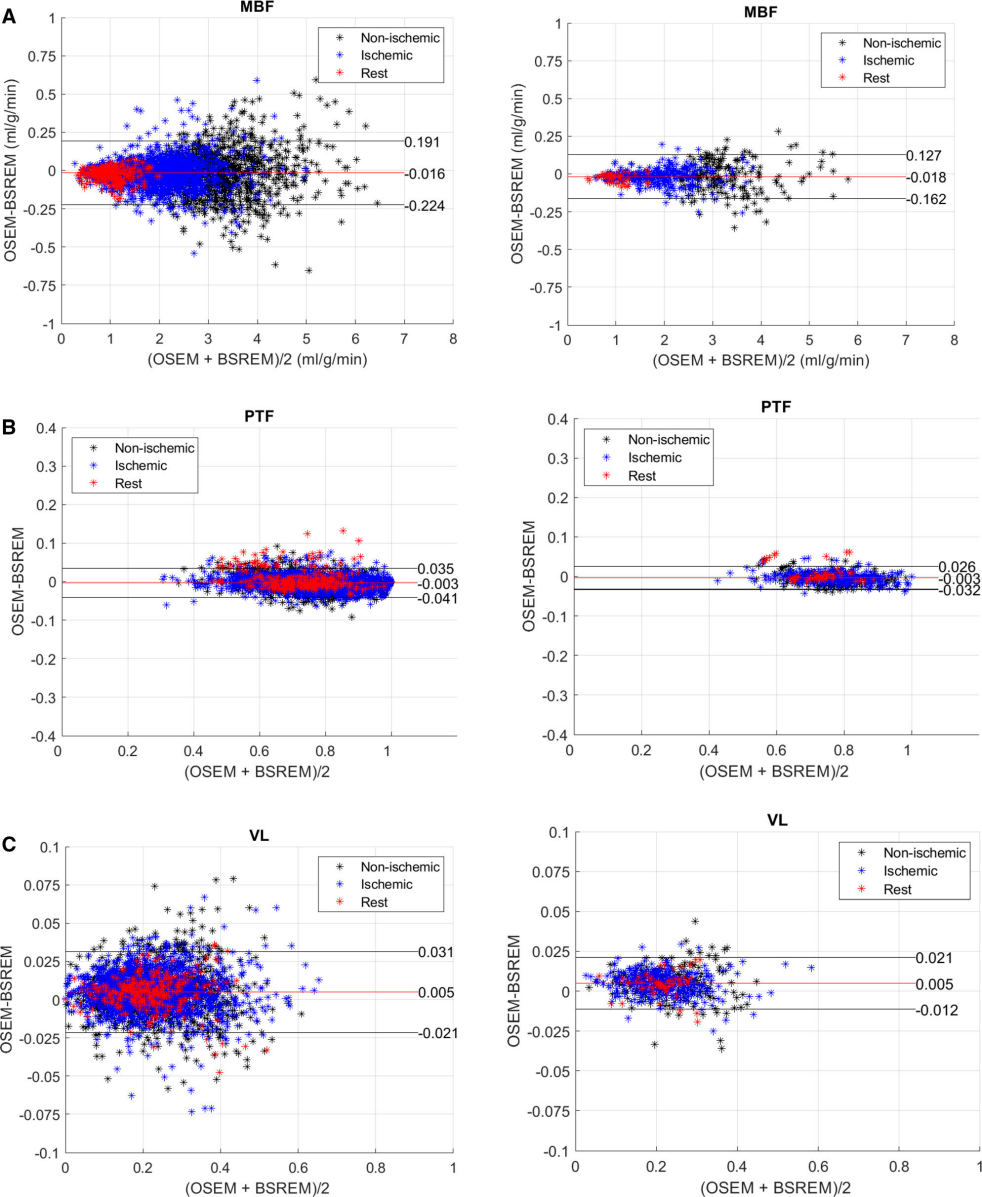
Bland–Altman scatter plots of segmental **a** MBF (ml⋅g^*−*1^⋅min^*−*1^), **b** PTF, and **c** VL obtained using BSREM and OSEM-TOF-PSF reconstructions. Results for 17 segments are shown in the left column and 3 segments (LAD, RCA and LCX) in the right column

Figure 3 shows Bland–Altman plots of segmental MBF, PTF, and VL obtained using OSEM-TOF-PSF and BSREM reconstructions. The mean bias between OSEM-TOF-PSF and BSREM is near zero for all parameters and segments. The Lines-of-Agreement (LoAs) are smaller than 0.3 ml⋅g^−1^⋅min^−1^ (Figure 3a), 0.05 (Figure 3b), and 0.04 (Figure 3c) for MBF, PTF, and VL in both 17- and 3-segment plots. Non-ischemic patients show largest variation between OSEM-TOF-PSF and BSREM compared to ischemic and rest subjects.

The 17- and 3 segment MBF polar maps appeared visually similar between OSEM-TOF-PSF and BSREM reconstructions.

OSEM-TOF-PSF classified 115 out of 177 subjects ischemic (MBF < 2.3 ml⋅g^−1^⋅min^−1^ in at least two contiguous segments) from the 17-segment polar maps. The corresponding number was 113 for the BSREM reconstruction. All patients were classified similarly from the 3-segment polar maps with both reconstructions.

**Figure 4:**
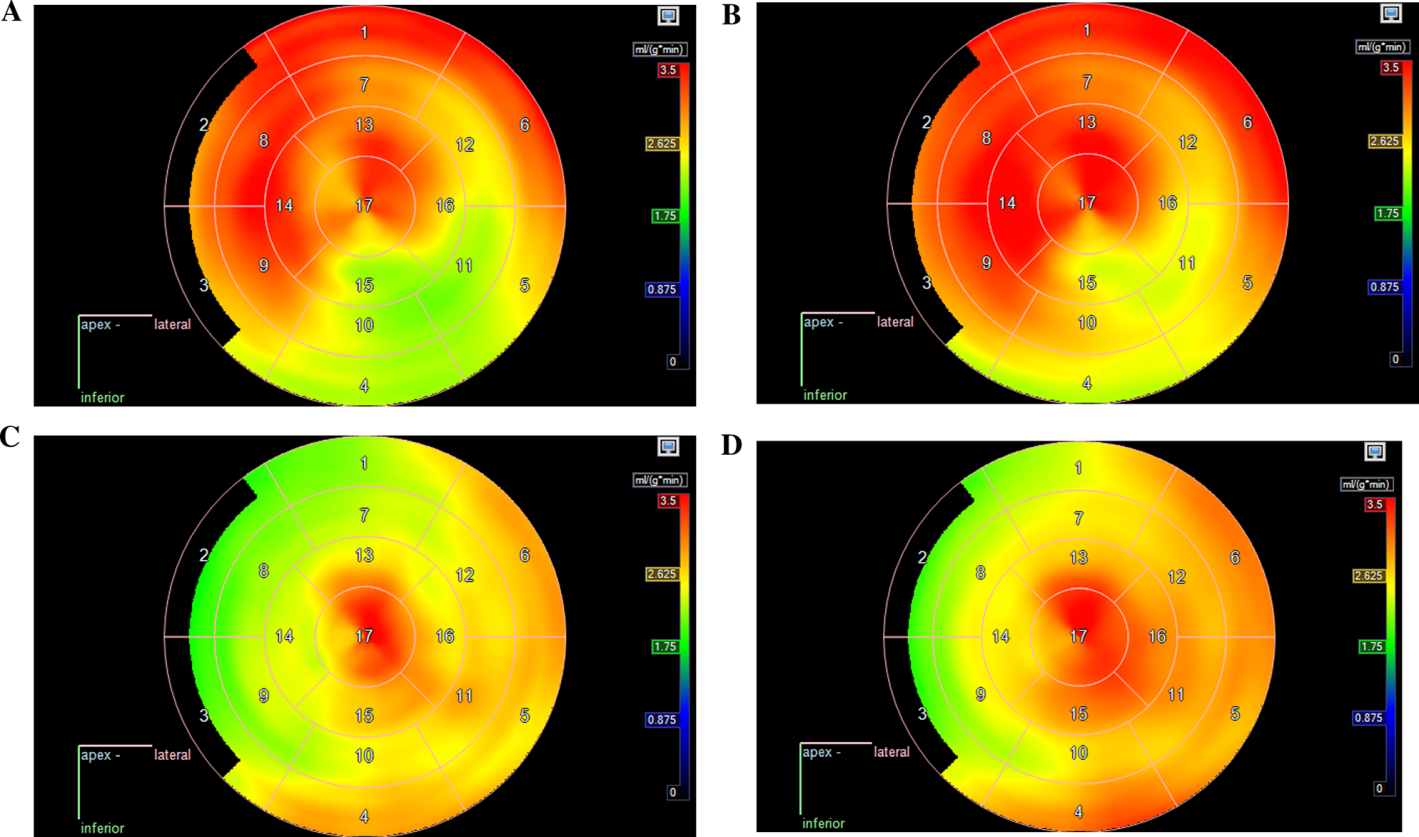
The 17-segment polar maps of the two subjects classified discordantly between OSEM-TOF-PSF and BSREM based on the definition by Danad et al.^5^ In **a** OSEM-TOF-PSF (1st patient) and **b** BSREM (1st patient), **c** OSEM-TOF-PSF (2nd patient) and **d** BSREM (2nd patient) reconstructions are presented. The patients were classified ischemic based on the OSEM-TOF-PSF reconstruction and non-ischemic based on the BSREM reconstruction

Figure 4 presents the 17-segment polar maps of the patients with discrepant classification between OSEM-TOF-PSF (Figure 4a, c) and BSREM (Figure 4b, d) based on the definition by Danad et al.^5^ The overall visual impression is similar for both patients. In one patient, OSEM-TOF-PSF classified four segments ischemic, whereas BSREM classified only one segment ischemic. In the other patient, OSEM-TOF-PSF classified five segments ischemic and BSREM two segments. MBF, PTF, and VL values for each segment are presented in Supplementary File I.

## Discussion

We performed a retrospective evaluation of the recently introduced BSREM reconstruction algorithm in comparison to the OSEM-TOF-PSF algorithm for the quantitation of myocardial perfusion using [^15^O]H_2_O MPI PET. Data from 177 subjects with suspected obstructive coronary artery disease undergone adenosine stress or rest-stress MPI were evaluated. Both algorithms provided similar absolute values across a wide range of MBF in ischemic myocardial regions and in the normal myocardium. Furthermore, classification of myocardial ischemia was highly concordant with BSREM and OSEM-TOF-PSF.

In general, BSREM showed the largest mean relative differences of 4%, 2%, and 6% for the stress studies and 7%, 4% and 6% for the rest studies in comparison to OSEM-TOF-PSF for MBF, PTF, and VL, respectively (Table 1). These values are similar to our previous study where the flow values differed 7% between the reconstructions on average on a dynamic PET flow phantom.^20^ Similarly, the segmental boxplots showed that BSREM produced no more than 5% (Figure 1a, b) difference to OSEM-TOF-PSF in terms of medians and quantiles, and no clear differences were noted in terms of absolute values (Figure 1c, d) in whole patient cohort. Furthermore, the R^2^ values from the correlation analysis were larger than 0.93 for all parameters and segments in the whole study population (Figure 2). What is more, patients classified non-ischemic showed larger variation between OSEM-TOF-PSF and BSREM reconstructions in Bland–Altman plots (Figure 3) compared to ischemic and rest subjects. However, we consider this variation negligible since the bias was very close to zero and the LoAs were smaller than 0.5 for all parameters across the group.

The BSREM-based classification was concordant to OSEM-TOF-PSF in 99% (175 out of 177) of subjects based on the threshold of ischemia that has been previously defined in Danad et al.^5^ BSREM classified the subjects ischemic and OSEM-TOF-PSF non-ischemic. However, the visual interpretation from the BSREM polar maps indicated only very small differences to OSEM-TOF-PSF for these subjects (Figure 4) caused by random noise and MBF variability close to threshold of ischemia.^5^ Ultimately, the time-activity-curves (TACs) (not shown here) were also similar for these subjects between OSEM-TOF-PSF and BSREM. The differences in the PTF and VL parameters between OSEM-TOF-PSF and BSREM were also small. The PTF parameter is defined as the fraction of tissue capable of rapidly exchanging [^15^O]H_2_O within a given volume of region of interest. Therefore, it plays a role in the differentiation of viable myocardium from infarct scar.^26^ Our study shows similar PTF values using either OSEM-TOF-PSF or BSREM reconstruction suggesting feasibility in assessment of myocardial viability. The VL parameter is not currently used in clinical interpretation but our study indicates it to remain similar between OSEM-TOF-PSF and BSREM.

BSREM reconstruction has been previously studied widely in the field of oncology with [^18^F]FDG, where superior standardized-uptake-values (SUVs) compared to OSEM-TOF-PSF have been demonstrated.^11–15^ Previously, O’Doherty et al. evaluated the MBF values in BSREM reconstruction in rest [^13^N]NH_3_ MPI for 21 subjects.^16^ The study showed that the resting MBF values are closely correlated (*P* > .95) across BSREM and other reconstructions.^16^ Similarly, Nordström et al*.* found only minor differences in MBF quantification between various reconstructions for [^15^O]H_2_O.^17^ The findings of our study complement these results, as no significant difference was measured between OSEM-TOF-PSF and BSREM (*P* > .05) in patients with suspected CAD studied at rest and during stress using a large patient group. However, no such studies have been performed for [^82^Rb], to the best knowledge of the authors.

In addition to reconstruction algorithms, other factors contribute to variability in quantification of MBF. El Fakhri et al. have studied the reproducibility of MBF quantitation with [^82^Rb] and [^13^N]-NH_3_.^27^ They reported test–retest repeatability of 16% for stress MBF. Similar test–retest repeatability has been observed with [^15^O]H_2_O PET.^28^ Nesterov et al. have studied intra- and inter-observer repeatability of MBF quantification with the Carimas software used in this study.^29^ They reported intra-observer difference of 9% and inter-observer difference of 10% for analysis of MBF.^29^ Moreover, Nordström et al. reported an intra-and inter-observer variability of less than few percent, which can be considered to be within the limits of the inherent uncertainty of MBF measurements in their study.^30^ As our largest difference for any parameter was 7% we consider that these results fall below the day-to-day and intra- and inter-observer uncertainty.

We have also shown that there is only a minor difference in measured MBF between BSREM and OSEM-TOF-PSF, similarly to very recent study of Nordström et al.^17^ This may be partly explained by [^15^O]H_2_O relying on wash out/k2-based flow estimates in contrast to other perfusion tracers relying on wash in/K1-based flow estimates, which also stabilizes MBF quantification across various reconstruction options such as changing the beta value.^17^ For PTF, an effect might be observed if the tissue fraction within a delineated VOI would change drastically between reconstructions. However, this would need to be systematically studied in detail. The results of the study of Nordström et al*.*^17^ would indicate that the effect of different reconstructions as well as different beta values to PTF are minimal.

Moreover, non-TOF and non-PSF algorithms were not compared in this study as we applied two state-of-the-art reconstructions available on a clinical PET/CT system. Previous studies performed by Armstrong et al*.*^31^ and Germino et al.^32^ have shown that incorporation of TOF and PSF is beneficial for MBF quantification. Armstrong et al. showed improvement in accuracy of MBF values when applying OSEM-TOF-PSF in 37 patients undergone [^82^Rb] PET perfusion imaging.^31^ In addition, Germino et al. showed that OSEM-TOF-PSF reconstruction reduces standard error of estimated kinetic parameters and improves quality of parametric [^82^Rb] and [^15^O]H_2_O images.^32^

In our study, small differences in MBF between OSEM-TOF-PSF and BSREM can be partly explained by incorporation of both TOF and PSF modeling in both OSEM-TOF-PSF and BSREM reconstructions. Image quality was also similar between the two algorithms, in line with O’Doherty et al. who reported similar [^13^N]NH_3_ image noise between OSEM-TOF and BSREM when beta values of 300 to 400 are used.

As a limitation to this study, only one analysis software (Carimas) with specific modeling for [^15^O]H_2_O^2–4^ was used. However, good comparability and reproducibility has been shown across various software for MBF analysis.^29^ Our results also complement the assumption of Norström et al.^17^ who applied a different analysis software for [^15^O]H_2_O, indicating that [^15^O]H_2_O quantification should be robust across various software.

Previous studies have also experimented several beta values (for example 100-1000 in Teoh et al.^15^) in BSREM reconstruction and their impact on the image quality. However, we applied only one beta value (350) due to the retrospective nature of our study. The beta value used in this study is routinely applied in MPI studies at our institute and is the default value set in the Discovery MI 20 4-ring system. This is close to the value of 400 recommended by Teoh et al.^15^ and value of 300 to 400 recommended by O’Doherty et al.^16^ Thus, the results of this study could be applied immediately in the clinical routine by using the default beta value (350) for the DMI system recommended by the manufacturer, enabling the use of BSREM for MBF quantification in [^15^O]H_2_O MPI PET.

## New knowledge gained

Myocardial perfusion imaging using [^15^O]H_2_O PET can be conducted applying either OSEM-TOF-PSF or BSREM (using a beta value of 350) reconstructions without any significant differences in terms of MBF, PTF and VL. The relative differences between OSEM-TOF-PSF and BSREM are smaller than 7%. Both reconstructions produce similar classification of ischemia.

## Conclusions

BSREM with beta value of 350 and OSEM-TOF-PSF algorithms provide similar quantification of MBF, PTF, and VL and result to similar classification of ischemia using [^15^O]H_2_O MPI PET in patients with suspected CAD.

## Supplementary Information

Below is the link to the electronic supplementary material.

Supplementary file1 (DOCX 168 kb)

Supplementary file2 (DOCX 26 kb)

## Abbreviations

MPI: Myocardial perfusion imaging
PET: Positron emission tomography
CAD: Coronary artery disease
MBF: Myocardial blood flow
PTF: Perfusable tissue fraction
VL: Vascular volume fraction
BSREM: Block-sequential-regularized-expectation-maximization
OSEM: Ordered-subset-expectation-maximization
TOF: Time-of-flight
PSF: Point-spread-function
