# Correction to: A retrospective evaluation of Bayesian-penalized likelihood reconstruction for [^15^O]H_2_O myocardial perfusion imaging

**DOI:** 10.1007/s12350-023-03253-z

**Published:** 2023-03-17

**Authors:** Reetta Siekkinen, Chunlei Han, Teemu Maaniitty, Mika Teräs, Juhani Knuuti, Antti Saraste, Jarmo Teuho

**Affiliations:** 1 Turku PET Centre, Turku University Hospital, Kiinamyllynkatu 4-8, 20520 Turku, Finland; 2 Turku PET Centre, University of Turku, Turku, Finland; 3 Department of Medical Physics, Turku University Hospital, Turku, Finland; 4 Heart Centre, Turku University Hospital, Turku, Finland; 5 Institute of Biomedicine, University of Turku, Turku, Finland

**Correction to: Journal of Nuclear Cardiology** https://doi.org/10.1007/s12350-022-03164-5

The original published Supplementary File I was incorrect and is replaced with the following Supplementary File: “12350_2022_3164_MOESM2_ESM”.

The original article has been corrected.

