# Supplementary material for: A retrospective evaluation of Bayesian-penalized likelihood reconstruction for [15O]H2O myocardial perfusion imaging
Source: J Nucl Cardiol. 2023 Jan 19;30(4):1602–12. doi: 10.1007/s12350-022-03164-5 (PMC10371909; doi:10.1007/s12350-022-03164-5)
Supplement: Supplementary file 1 — Supplementary file1 (DOCX 168 kb) [file 12350_2022_3164_MOESM1_ESM.docx]

Additional information:

**A retrospective evaluation of Bayesian-penalized likelihood reconstruction for [^15^O]H2O myocardial perfusion imaging**

Reetta Siekkinen^1,2,3^, Chunlei Han^1^, Teemu Maaniitty^1^, Mika Teräs^3,5^, Juhani Knuuti^1,2^, Antti Saraste^1,2,4*^, Jarmo Teuho^1,2*^

^
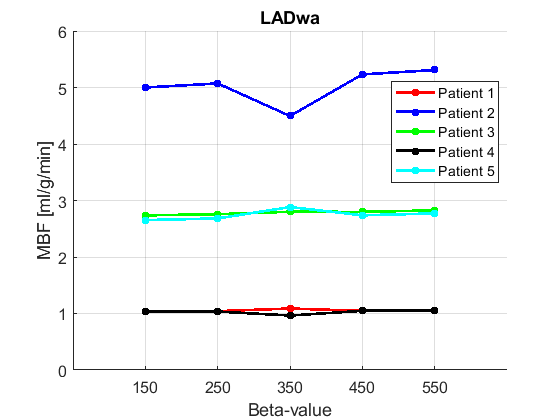

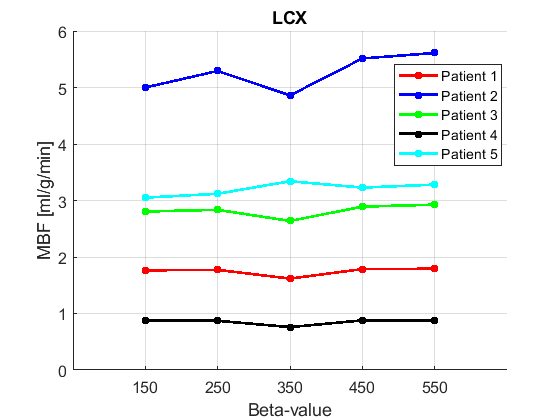

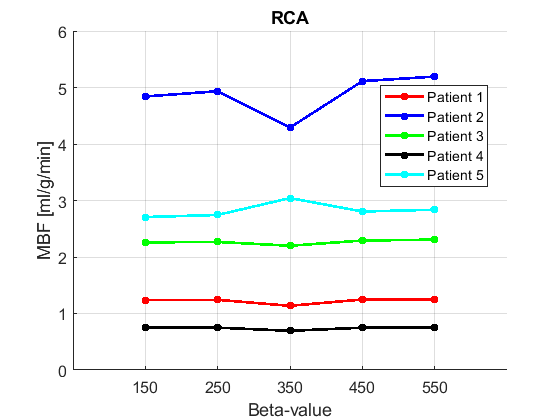
^

Figure 1. Myocardial blood flow (MBF) values calculated for BSREM with different beta-values (150, 250, 350, 450, 550) for each patient and three coronary territories (LAD, LCX, and RCA). The lineplots show differences in MBF for all beta-valuesthat are very small.


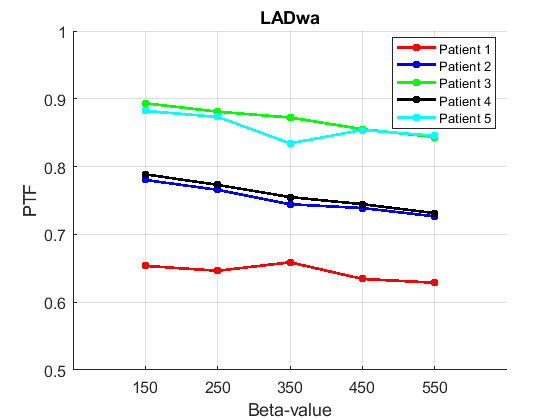

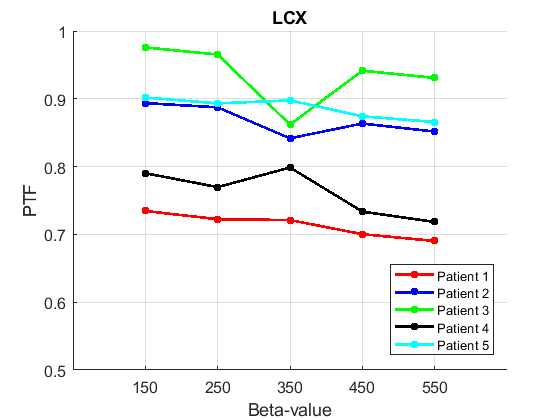

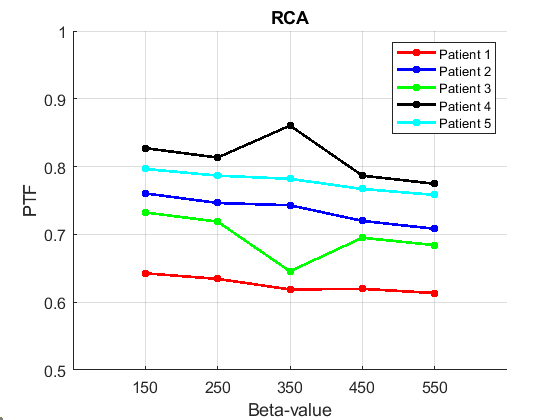


Figure 2. Perfusable tissue fraction (PTF) values calculated for BSREM with different beta-values (150, 250, 350, 450, 550) for each patient and three coronary territories (LAD, LCX, and RCA). The lineplots show only small differences in PTF for all beta-values.

^
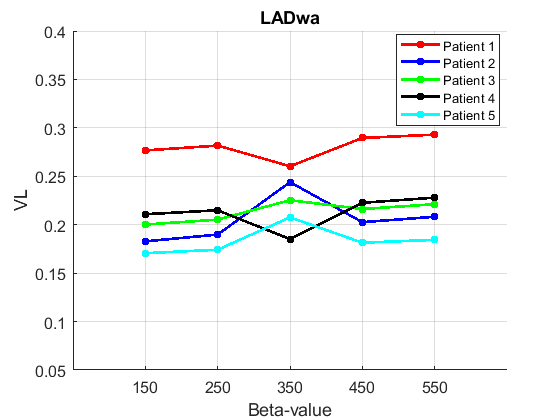

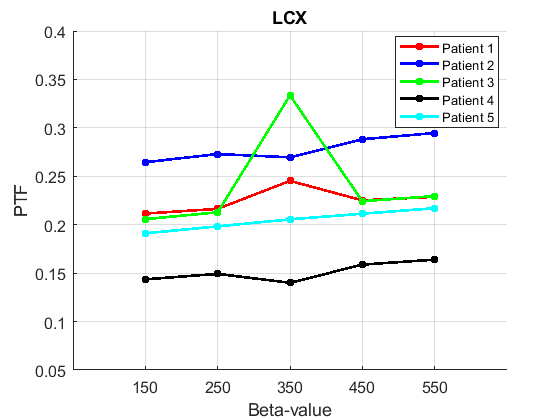

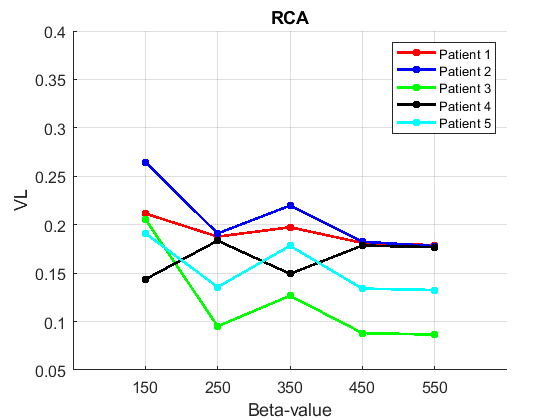
^

Figure 3. VL values calculated for BSREM with different beta-values (150, 250, 350, 450, 550) for each patient and three coronary territories (LAD, LCX, and RCA). The lineplots show the difference in VL for all beta-values. The differences are very small.

Table I. Mean±SD MBF, PTF, and VL values in five patients calculated in BSREM reconsctructions using different beta-values (150, 250, 350, 450, 550) in three coronary territories (LAD, LCX and RCA). The largest SD calculated is 0.27 ml/g/min for MBF, 0.02 for PTF, and 0.06 for VL indicating that differences between different beta-values are very small.

|  | 1 |  | 2 |  | 3 |  | 4 |  | 5 |  |
| --- | --- | --- | --- | --- | --- | --- | --- | --- | --- | --- |
|  | **Mean±SD** |  | **Mean±SD** |  | **Mean±SD** |  | **Mean±SD** |  | **Mean±SD** |  |
| **MBF** |  |  |  |  |  |  |  |  |  |  |
| LADwa | 1,0 | 0,00 | 5,2 | 0,14 | 2,8 | 0,04 | 1,0 | 0,01 | 2,7 | 0,05 |
| LCX | 1,8 | 0,01 | 5,4 | 0,27 | 2,9 | 0,05 | 0,9 | 0,01 | 3,2 | 0,10 |
| RCA | 1,2 | 0,01 | 5,0 | 0,16 | 2,3 | 0,02 | 0,8 | 0,00 | 2,8 | 0,06 |
| **PTF** |  |  |  |  |  |  |  |  |  |  |
| LADwa | 0,6 | 0,01 | 0,8 | 0,02 | 0,9 | 0,02 | 0,8 | 0,03 | 0,9 | 0,02 |
| LCX | 0,7 | 0,02 | 0,9 | 0,02 | 1,0 | 0,02 | 0,8 | 0,03 | 0,9 | 0,02 |
| RCA | 0,6 | 0,01 | 0,7 | 0,02 | 0,7 | 0,02 | 0,8 | 0,02 | 0,8 | 0,02 |
| **VL** |  |  |  |  |  |  |  |  |  |  |
| LADwa | 0,3 | 0,01 | 0,2 | 0,01 | 0,2 | 0,01 | 0,2 | 0,01 | 0,2 | 0,01 |
| LCX | 0,2 | 0,01 | 0,3 | 0,01 | 0,2 | 0,01 | 0,2 | 0,01 | 0,2 | 0,01 |
| RCA | 0,2 | 0,01 | 0,2 | 0,04 | 0,1 | 0,06 | 0,2 | 0,02 | 0,1 | 0,03 |
