# Supplementary material for: A retrospective evaluation of Bayesian-penalized likelihood reconstruction for [15O]H2O myocardial perfusion imaging
Source: J Nucl Cardiol. 2023 Jan 19;30(4):1602–12. doi: 10.1007/s12350-022-03164-5 (PMC10371909; doi:10.1007/s12350-022-03164-5)
Supplement: Supplementary file 2 — Supplementary file2 (DOCX 26 kb) [file 12350_2022_3164_MOESM2_ESM.docx]

**Supplementary File I:**

**A retrospective evaluation of Bayesian-penalized likelihood reconstruction for [^15^O]H2O myocardial perfusion imaging**

Reetta Siekkinen^1,2,3^, Chunlei Han^1^, Teemu Maaniitty^1^, Mika Teräs^3,5^, Juhani Knuuti^1,2^, Antti Saraste^1,2,4*^, Jarmo Teuho^1,2*^

Affiliations:

1: Turku PET Centre, Turku University Hospital, Turku, Finland

2: Turku PET Centre, University of Turku, Turku, Finland

3: Department of Medical Physics, Turku University Hospital, Turku, Finland

4: Heart Centre, Turku University Hospital, Turku, Finland

5: Department of Biomedicine, University of Turku, Turku, Finland

*: Equal Contribution

Correspondence: reetta.siekkinen@tyks.fi

Two subjects were classified differently between BSREM and OSEM-TOF-PSF. OSEM-TOF-PSF classified the subjects ischemic and BSREM non-ischemic. The first subject was determined ischemic based on segments 4, 10 and 15 with OSEM-TOF-PSF. The second patient was classified ischemic based on segments 1, 2, 3, 8, and 9 with OSEM-TOF-PSF.

In the first subject, MBF values for segments 4, 10, and 15 were 2.1749 ml/g/min, 2.2958 ml/g/min, and 2.2311 ml/g/min for OSEM-TOF-PSF, and 2.2890 ml/g/min, 2.6924 ml/g/min, and 2.4308 ml/g/min for BSREM. Thus, MBF in segments 10 and 15 was above the ischemic cut-off value with the use of BSREM.

PTF in these segments of the first subject were 0.5638, 0.7182, and 0.6169 for OSEM-TOF-PSF and 0.5575, 0.7203, and 0.6521 for BSREM. VL values were 0.3088, 0.3622, and 0.4018 for OSEM-TOF-PSF, and 0.3095, 0.3312, and 0.3567 for BSREM, respectively.

In the second subject, MBF values of segments 1, 2, 3, 8, and 9 were 2.1441 ml/g/min, 1.7641 ml/g/min, 1.7329 ml/g/min, 2.1135 ml/g/min, and 2.1977 ml/g/min for OSEM-TOF-PSF, and 2.3841 ml/g/min, 1.8139 ml/g/min, 1.6856 ml/g/min, 2.3548 ml/g/min, and 2.3060 for BSREM. Thus, MBF in segments 1, 8 and 9 was above the ischemic threshold with the use of BSREM.

PTF values in these segments of the second subject were 0.4984, 0.5159, 0.4990, 0.6270, and 0.6655 for OSEM-TOF-PSF and 0.4617, 0.4793, 0.4492, 0.6211, and 0.6254 for BSREM. The VL values were 0.3984, 0.4491, 0.4462, 0.3818 and 0.3653 for OSEM-TOF-PSF, and 0.4273, 0.4884, 0.4900, 0.4007, and 0.4017 for BSREM, respectively.

In summary, 5 segments in 2 subjects were classified differently with OSEM-TOF-PSF and BSREM.
